# Alignment of Short Reads: A Crucial Step for Application of Next-Generation Sequencing Data in Precision Medicine

**DOI:** 10.3390/pharmaceutics7040523

**Published:** 2015-11-23

**Authors:** Hao Ye, Joe Meehan, Weida Tong, Huixiao Hong

**Affiliations:** Division of Bioinformatics and Biostatistics, National Center for Toxicological Research, U.S. Food and Drug Administration, 3900 NCTR Road, Jefferson, AR 72079, USA; E-Mails: hao.ye@fda.hhs.gov (H.Y.); joe.meehan@fda.hhs.gov (J.M.); weida.tong@fda.hhs.gov (W.T.)

**Keywords:** precision medicine, next-generation sequencing, genetic variants, alignment, short reads

## Abstract

Precision medicine or personalized medicine has been proposed as a modernized and promising medical strategy. Genetic variants of patients are the key information for implementation of precision medicine. Next-generation sequencing (NGS) is an emerging technology for deciphering genetic variants. Alignment of raw reads to a reference genome is one of the key steps in NGS data analysis. Many algorithms have been developed for alignment of short read sequences since 2008. Users have to make a decision on which alignment algorithm to use in their studies. Selection of the right alignment algorithm determines not only the alignment algorithm but also the set of suitable parameters to be used by the algorithm. Understanding these algorithms helps in selecting the appropriate alignment algorithm for different applications in precision medicine. Here, we review current available algorithms and their major strategies such as seed-and-extend and q-gram filter. We also discuss the challenges in current alignment algorithms, including alignment in multiple repeated regions, long reads alignment and alignment facilitated with known genetic variants.

## 1. Introduction

Under the “one-size fits all” therapy model in conventional medicine, certain medical interventions can be more effective or cause fewer side effects for some patients than for others. Therefore, it is important to identify the potential patients who are more or less likely to benefit from the intervention. Precision medicine, or personalized medicine, has been proposed as a modernized and promising medical strategy, which emphasizes prevention and treatment strategies that take individual variability into account [1]. Thus, suitable individuals can receive proper treatment based on their individual genetic makeups. Although many factors, including environment, lifestyle and medical history contribute to the differences in treatment of drugs among individuals, genomics provides the most comprehensive genetic characteristics of each individual and is often believed to be the leading driver of precision medicine [2].

Genetic biomarkers play key roles in implementation of precision medicine. There has been much effort to advance biomarker discovery and application in regulatory science [3,4,5,6,7,8]. Emerging technologies have been used for biomarker development [9]. Many genetic biomarkers that are used in clinical practice and drug development were identified through genome-wide association studies (GWAS) using genotyping microarray technology [10]. There are some sources of microarray genotyping errors [11], including batch effect [12,13,14,15] and variation in genotype calling algorithms [16,17,18], which are considered part of the reason for why GWAS have not fully satisfied the expectations of scientists to completely decipher the human genetic architecture. Recently, next-generation sequencing (NGS) technologies have emerged as the most popular tools for deciphering human genetic variations [19], profiling miRNA [20], and identifying genetic biomarkers for clinical diagnosis [21] and prognosis [22]. Quality control is important for better utilization of NGS data [23] and proteomics data [24].

Scientists have already launched several large human genetics projects in order to obtain a detailed catalogue of human genetic variation, such as the 1000 genomes project [25] and the Yan Huang project [26]. The illumina estimation indicates that, as of 2014, ~228,000 human genomes had been completely sequenced in the world [27]. The number of human genomes sequenced is expected to double about every 12 months, reaching ~1.6 million genomes by 2017 [28]. With the cost of human sequencing having dramatically dropped from $3 billion for the Human Genome Project to $1000 currently achieved by illumina X Ten system, the bottleneck of genomics has shifted from sequencing experiments to analyzing and interpreting the sequencing data [29,30].

**Figure 1:**
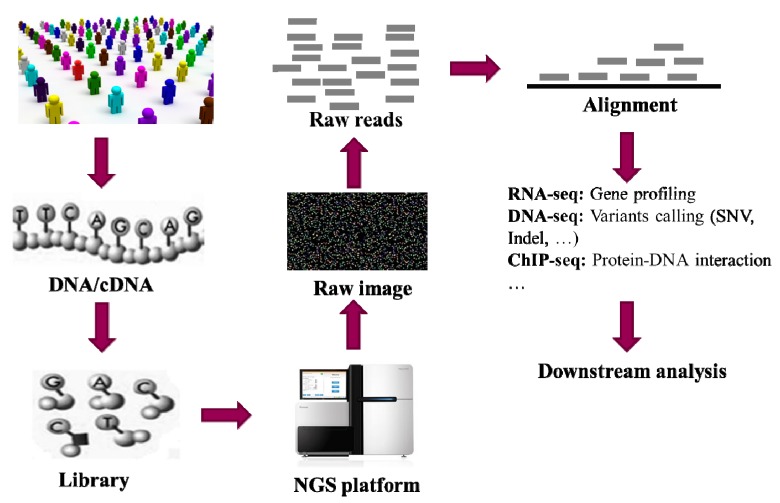
A brief flow chart of genetic studies using NGS. In the first step, DNA or cDNA samples are extracted from the cells of individuals. Then each of the samples is cleaved into small fragments and PCR is carried out to build the library by amplifying each of the small fragments. The library is then sequenced using the NGS platform. The original output from the NGS platform is a set of images. Thereafter, a base-calling algorithm is used to processing the images and output the so-called “raw reads”. An alignment algorithm is then used to align the millions of short reads onto the reference human genome followed up by genetic variants detection for downstream analysis in the genetics studies.

Figure 1 gives a typical workflow in genetic studies using NGS (next-generation sequencing) technology, including DNA extraction, DNA library building, sequencing, alignment, genetic variants detection and downstream data analysis. Millions of raw reads with a length from 175 to 300 bp are usually generated from current NGS platforms (Table 1). However, the latest developed PacBio RII platform generates very long reads (up to 40k bp). With the raw reads, data analysis methods are used to detect the genetic variants in the samples. Alignment of these raw reads into a reference genome is the first and essential step in almost all applications, such as genetic variants detection, methylation patterns profiling (MeDIP-Seq), protein-DNA interactions mapping (ChIP-Seq), and differentially expressed gene identification (RNA-Seq). All these applications require aligning large quantities of short reads to the human genome in a reasonably short time. Generally, the alignment process should quickly determine the correct position of the reads in the genome in consideration of sequencing error and heterozygous variation [31]. To keep pace with the high-speed development of sequencing technologies, many alignment algorithms and tools have been developed in the last few years. Table 2 summarizes some popular alignment algorithms and software tools, without the intension of giving a complete list.

In this article, we review the basic strategies frequently used in current alignment algorithms. We also discuss the challenges in alignment of short reads.

**Table 1:** The most frequently used NGS platforms *.

Platform Name	Illumina HiSeq 2500	Ion Torrent-Proton II	PacBio RS II	OxFord Nanopore Minion
Instrument	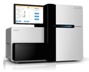	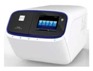	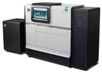	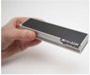
Cost (USD) **	690 k	224 k	695 k	1 k ***
Reagent cost Per run/per GB	4126/45.84	1000/20.41	100/1111.11	900/1000
Reads per run	300 millions	280 millions	0.03 millions	0.1 millions
Average Read length	2 × 150 bp	175 bp	14,000 bp	9,000 bp
Run time	10 h	5 h	2 h	6 h
Major errors	substitution	indel	indel	deletion
Error rate (%)	0.1	1	1	4
Amplification	bridgePCR	emPCR	none, SMS	none, SMS
Advantage	low cost per GB; high output	low cost	long reads; no amplification bias	long reads; no amplification bias
Disadvantage	high cost	homopolymer errors	low throughput; high cost	high error rate

* Sources: http://www.molecularecologist.com/next-gen-fieldguide-2014/ and websites of the companies; ** Sources: http://www.molecularecologist.com/next-gen-table-3a-2014/; *** Accessing fee. Sources: https://www.nanoporetech.com/products-services/minion-mki.

**Table 2:** Alignment algorithms and software tools.

Name	Website	Reference	Remark
SOAP *	soap.genomics.org.cn	[32,33,34,35]	k-mer inexact match seed; support at most 3 mismatches; GPU calculation supported
CUSHAW ^$^	cushaw3.sourceforge.net/homepage.htm#downloads	[36,37,38,39]	k-mer inexact match, maximal exact match and hybrid seeds; GPU supported
Bowtie ^&^	bowtie-bio.sourceforge.net	[40,41]	k-mer inexact match seed; high speed; double-index; up to 3 mismatches
BWA	bio-bwa.sourceforge.net	[42,43]	k-mer inexact match and maximal exact match seed
GASSST	www.irisa.fr/symbiose/projects/gassst/	[44]	k-mer exact match seed; it currently has been tested for reads up to 500 bp
GNUMAP	dna.cs.byu.edu/gnumap/	[45]	k-mer exact match seed; probabilistically mapping reads to repeat regions
MOSAIK	gkno.me/pipelines.html#mosaik	[46]	k-mer exact match seed
NextGenMap	cibiv.github.io/NextGenMap/	[47]	q-gramq-gram filter; GPU calculation supported
QPALMA	www.raetschlab.org/suppl/qpalma	[48]	k-mer inexact match; incorporate read quality score and splice site
RMAP	rulai.cshl.edu/rmap/	[49,50]	k-mer inexact match seed; 10 mismatches allowed; incorporate read quality score
Segemehl	www.bioinf.uni-leipzig.de/Software/segemehl/	[51]	k-mer inexact match seed; enhanced suffix arrays
SeqMap	www-personal.umich.edu/ ~jianghui/seqmap/	[52]	k-mer inexact match; support windows, linux, Mac OS
Stampy	www.well.ox.ac.uk/project-stampy	[53]	k-mer inexact match; support up to 30 bp indels in paired-end reads alignment
Cloudburst	sourceforge.net/projects/cloudburst-bio/	[54]	Highly sensitive read mapping with MapReduce.
drFAST	drfast.sourceforge.net/	[55]	k-mer inexact match; specially designed for better delineation of structural variants
BFAST	sourceforge.net/projects/bfast/	[56]	k-mer spaced seeds
MAQ	maq.sourceforge.net	[57]	k-mer spaced seeds; incorporate quality scores of reads in alignment
MOM	go.vcu.edu/mom	[58]	k-mer spaced seeds; unlimited mismatches in the 3′ and 5′ flanking regions.
PASS	pass.cribi.unipd.it	[59]	k-mer spaced seeds; implemented in C++ and supported on Linux and Windows
PerM	code.google.com/p/perm/	[60]	k-mer spaced seeds; 9 mismatches are allowed
SHRiMP2	compbio.cs.toronto.edu/shrimp/	[61,62]	combined k-mer spaced seeds and q-gram filter
ZOOM	www.bioinfor.com/zoom/general/overview.html	[63]	k-mer spaced seeds; tolerate 2 mismatches by default
BarraCUDA	seqbarracuda.sourceforge.net/	[64]	Incorporate GPU to speed up BWA
GEM	gemlibrary.sourceforge.net/	[65]	q-gram filter
MPSCAN	www.atgc-montpellier.fr/mpscan/	[66]	q-gram filter; support Windows, linux, Mac OS
ERNE	iga-rna.sourceforge.net/	[67]	long gap support; Works on Windows, Mac OS X, linux
SARUMAN	www.cebitec.uni-bielefeld.de/ brf/saruman/saruman.html	[68]	k-mer inexact matched seed; support GPU calculation
LAST	last.cbrc.jp/	[69]	adaptive seed
Genalice MAP	www.genalice.com/product/genalice-map/	NA	cloud calculation; High sensitivity for SNPs and long INDELS
Novoalign	www.novocraft.com/	NA	support up to 7 and 16 mismatches in single-end and pair-end reads.
PRIMEX	bioinformatics.cribi.unipd.it/primex	[70]	k-mer inexact match seed; written in C++; lookup table and server functionality
SOCS	solidsoftwaretools.com/gf/project/socs/	[71]	good at align CpG methylation-enriched reads
SToRM	bioinfo.lifl.fr/yass/iedera_solid/storm/	[72]	doesn’t support pair-end reads
iSAAC	https://github.com/sequencing/isaac_aligner	[73]	k-mer inexact match seed; high speed
RazerS	www.seqan.de/projects/razers/	[74]	q-gram filter; support Windows, linux, Mac OS X
SSAHA2	www.sanger.ac.uk/resources/software/ssaha2/	[75]	k-mer inexact match seed; support various output formats
UGENE	ugene.unipro.ru/	[76]	works on Windows, linux and Mac OS X

* Include SOAP, SOAP2, SOAP3 and SOAP3-dp; ^$^ Include CUSHAW (k-mer inexact match seed), CUSHAW2 (maximal exact match seed) and CUSHAW3 (hybrid seeds); ^&^ Include Bowtie and Bowtie2. NA: commercial software, no reference available.

## 2. Strategies of Current Alignment Algorithms

In theory, a read can be successfully aligned onto a reference genome by applying a series of insertions, deletions, and substitutions. An alignment algorithm assigns a score to the alignment of a short read onto a reference to estimate how well they align. The score is used to identify the optimized location of the read in the reference genome. A good alignment algorithm is able to map reads onto a reference genome rapidly and accurately. Currently, most alignment algorithms utilize two major strategies: seed and extend and q-grams filter. In addition, the index methods that are used to memory-efficiently organize the reference genome and short reads are different among the alignment algorithms. Hash-table data structure was initially designed to scan and index sequence (raw reads or reference) in the first wave of alignment programs such as MAQ and SOAP [32]. However, Borrows–Wheeler Transformation (BWT) based FM-index was adopted by subsequently developed alignment algorithms such as BOWTIE [40], BWA [42] and SOAP2 [33]. Compared with the large memory usage in a hash-table based index, BWT based FM-index could index the human genome in less than 5.4 GB of memory [77]. It is interesting to note that the final index for the human genome used by BWA is approximately 2.3 GB in size. Although such index methods are important components in alignment algorithms, in this review, we will only focus on the two basic alignment strategies mentioned above. For each of the two strategies, only a few popular alignment algorithms will be selected for detailed illustration.

### 2.1. Seed-and-Extend Strategy

**Figure 2:**
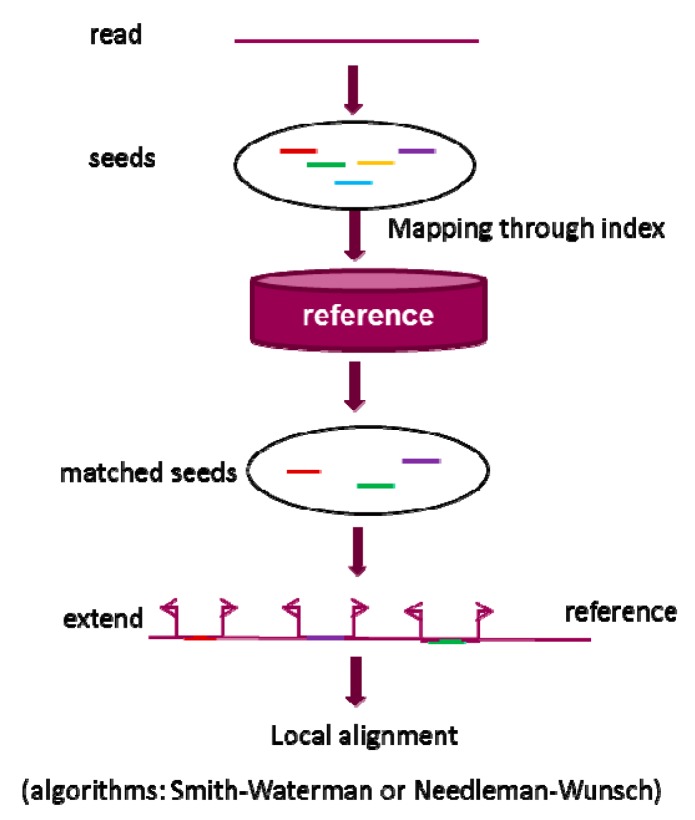
A brief workflow of seed-and-extend strategy in alignment. Generally, the strategy can be divided into three steps: (1) generate raw seed from a read; (2) identify the matched seed; and (3) extend the matched seed and do the local alignment through standard dynamic programming algorithms based on Needleman–Wunsch (NW) [78] algorithm or Smith–Waterman (SW) algorithm [79].

Seed-and-extend strategy is based on the observation that a good alignment should contain exact or inexact short matches between two sequences. Figure 2 shows a general view of seed-and-extend strategy that contains four steps: seed generation, seed mapping, extending each matched seed, and alignment of the read to the reference sequence. Seeds are the shorter sequences extracted from a read and can be generated using different methods. For example, k-mer seeds are generated by sliding a window of length *k* over the read. The seeds that exactly match a reference sequence can be identified through a mapping process facilitated by an index method. Each of the exactly matched seeds is then extended on both right and left direction under certain constraints such as maximum mismatches and length of indels. Standard dynamic programs, based on Needleman–Wunsch (NW) [78] algorithm or Smith–Waterman (SW) algorithm [79], are implemented to do the final alignment. Seed extension is usually more time-consuming than seed generation and final mapping, especially when the majority of the exacted mapped seeds cannot be completely extended to accomplish the alignment on the reference sequence. Therefore, seed filtration strategies are frequently used before extension. In addition, the seed length used by an alignment program has a substantial impact on its performance. Shorter seeds increase sensitivity, whereas longer seeds increase speed.

#### 2.1.1. k-mer Exact Match Seed

A k-mer exact match seed is a shorter sequence of *k* bases that exactly matches with a reference sequence. The strategy to use k-mer exact match seeds was first utilized in BLAST [80]. In brief, k-mer (default 11-mer) seeds that exactly match certain regions in reference genome are identified. Those seeds are then extended to match the reference genome without gaps. The final alignment results are generated by the SW modification on the extending sequence. Several improvements have been made on the extending and dynamic programming used in this strategy. For example, GNUMAP [45] incorporates the base quality of a read into NW algorithm to improve the alignment accuracy and uses 9-mer seeds to initiate the mapping process.

#### 2.1.2. k-mer Inexact Match Seed

A k-mer inexactly match seed is generated from a read based on pigeonhole principle. The strategy using k-mer inexactly match seeds supports mismatches and indels in mapping. The rationale behind the strategy using k-mer inexactly match seeds is that if m bases are allowed to mismatch between a read and a reference sequence, the read is has *n* bases, and the read can be chopped into non-overlapping k-mers (*k* = *n*/(*m* + 1)), at least one exact match k-mer seed exists. The k-mer inexact match has been utilized in many alignment algorithms.

SOAP [32] splits a read into fragments, based on the number of mismatches allowed (default five), to implement the strategy using inexact match seeds. Newer SOAP versions improve the alignment speed but use the same seeding strategy. SOAP2 [33] speeds up the mapping process using a reference sequence that is indexed by the combination of BWT and hash table. The graphics processing unit (GPU) is incorporated by SOAP3 to facilitate parallel calculation [34]. More recently, SOAP3-dp [35] utilized dynamic programing and bi-directional BWT to reduce unsuccessful extending of the seeds with multiple locations in the reference.

Bowtie [40,41] generates k-mer inexact match seeds with at most three (default two) mismatches in the high-quality end of a read (default: the first 28 bp in the read). The “double indexing” technology is the one of major contributions to the high speed of Bowtie. One is called “forward” index, which contains the BWT of a reference sequence, and another is referred as “mirror” index, which is composed of the BWT of the reversed reference sequence. Using double indexes, exactly matched seeds can be quickly identified. Bowtie uses a cutoff, the maximum acceptable quality score (default 70), to determine whether extension of a read continues. If the alignment has a quality score larger than the cutoff, the extension on the matched seed is stopped. This quality score constraint helps remove a lot of matched seeds for continuous extension alignment as early as possible.

BWA [42,43] generates k-mer inexactly match seeds with a default of two mismatches allowed in each seed. Seeds are efficiently mapped to a reference genome, facilitated by a special index structure called prefix directed acyclic word graph (DAWG) [81]. DAWG represents the set of all substrings that are extracted from a string. In BWA, alignment speed is improved by reducing unnecessary seed extension for highly repetitive sequences. In brief, BWA heuristically identifies and discards seed extensions using a criterion in which the length of the overlapped region is shorter than the length of any previous successfully aligned regions in the reference genome. BWA only reports the alignments that are largely non-overlapped with the query sequence instead of giving all the local alignments.

#### 2.1.3. k-mer Spaced Seed

Generally speaking, a seed allowing internal mismatches is called a spaced seed [82]. For example, in a 5-mer spaced seed “10110”, “1” indicts the position in which the base of a seed has to match with a reference sequence and “0” means the position in which the base of a seed is allowed to mismatch with a reference sequence. k-mer spaced seed was first introduced and proved to improve sensitivity of k-mer exact match in DNA homology searching by Ma [83].

RMAP [49,50] integrated base-calling quality scores to improve sensitivity in both seed mapping and extension processes. The quality scores from base-calling were used to weigh the penalties for mismatches at different positions. A mismatched base at a position with a lower quality score than the base-calling in a read is penalized less. Utilization of base-calling scores in alignment displayed high sensitivity in mapping of the bases for which the base caller has difficulties to call and thus gives low scores to the called bases.

MAQ [57] uses k-mer exact match seeds for mapping in the first step. When seeds fail to be exactly matched onto a reference sequence, MAQ generates 6-mer spaced seeds in which two or fewer mismatches are allowed in the first 28 bp for a read. This strategy saves a lot of time in seeds generation and mapping. In seed extension process, MAQ assigns each individual alignment a phred-scaled quality score (capped at 99) that is used to measure the probability that the true alignment is not the one found by MAQ. The larger phred the scaled quality score the better the alignment. The phred-scaled quality score is calculated as the sum of qualities of mismatched bases over the whole length of a read. When a read can be aligned equally well to multiple positions, MAQ randomly pick one position and gives it a mapping quality score zero.

#### 2.1.4. Maximum Extend Match (MEM) Seed

MEM [84] is an exact match between two strings that cannot be extended in either direction without allowing a mismatch. Compared to fixed-length seeds (seed length is predefined) mentioned above, the variable seed length is the key feature of MEM seed. MEM reduces the number of mapping positions of each seed onto a reference genome. Alignment speed using MEM seeds is improved because invalid seed extensions are prevented. The efficiency of generating MEM seeds plays a key role in an alignment algorithm using MEM seeds. In general, indexing a sequence in a full-text suffix tree is the frequently used strategy in detecting MEMs [85,86]. The obvious drawback of using full-text suffix tree is the large memory usage. Recently developed methods improved index structure to reduce the memory usage. Enhanced suffix array (ESA) was first introduced as a space-sparse suffix array to replace full-text index in suffix tree [85] and can be used to find MEMs with much less memory usage [87]. Khan [84] developed an algorithm to generate a special sparse suffix array that stores every k-th position of the text. In contrast to a full-text index that stores every position of the text, a sparse suffix array uses much less memory. Fernandes developed slaMEM to detect MEMs [88]. The slaMEM algorithm uses a new index structure called longest common prefix (LCP) array and the backward search method of the FM-index and achieves a good tradeoff between mapping speed and memory usage. More recently, E-MEM was developed by Nilesh to decipher MEMs in large genome sequences. E-MEM uses much less memory and is highly amenable to parallelization. It has been reported that all MEMs of minimum length 100 between whole human and mouse genomes could be calculated within 10 min on a 12-core machine, using 2 GB of memory [89].

BWA-MEM is the latest developed algorithm in BWA software for sequence alignment. BWA-MEM utilizes a new index structure called FMD-index in which both forward and reverse strand DNA sequences are indexed. It can efficiently facilitate detection of all MEMs between a read and a reference sequence. The super-maximal exact matches (SMEMs) are the matches that are not contained in any other MEMs of the read and are chosen for seed mapping and extension. Using SMEMs saves a lot of alignment time by reducing most invalid extensions of all other MEMs in the read. If a read cannot be aligned to a reference sequence by extension of the SMEMs, BWA-MEM uses a re-seeding process to generate new seeds for mapping and extension. Specifically, when the length of a SMEM is larger than 28 bp (default), the longest MEM seed which covers the middle of the SMEM in this read is used to initialize re-seed. In seed extension, BWA-MEM stops an extension at a certain point if the difference between the best alignment score in the extension and the score at that point is larger than a predefined value that is further adjusted by number of gaps in the alignment. When an extension reaches the whole read, this algorithm accepts the alignment as a successful one mapping between the read and the reference sequence if the best improvement in alignment score in the extension is larger than a predefine value.

CUSHAW2 [38] is another software package that uses MEM seeds to initiate alignment. MEM seeds are detected from the FM-index of a read or a reference sequence. An important parameter, Q, indicating the minimum seed length is used to filter MEM seeds to avoid invalid extensions. Specifically, the default Q is set at 16, 22 and 35 for the read length of 100, 200 and 500, respectively. Users can set Q. Parallelization with GPU is implemented in CUSHAW2.

#### 2.1.5. Adaptive Seed

Adaptive seed is the shortest sub-sequence in a read that exactly matches a reference sequence with a mapping frequency less than a predefined value. In contrast to fixed-length seeds, the length of an adaptive seed is not fixed, but determined through analyzing the mapping of possible seeds to the reference genome. To identify the adaptive seed for read, seeds with different lengths are generated at first. The frequency of mapped positions in the reference sequence for each of the generated seeds is then calculated. The seed that is the shortest among the seeds that have a mapping frequency less than a predefined value is selected as the adaptive seed for extension. Several algorithms have been developed for efficient identification of adaptive seeds.

LAST [69] was proposed by Kielbasa to reduce the redundancy of seeds in identification of adaptive seed. This algorithm selects the shortest seed among the seeds that exactly map to the reference sequence starting at the same position. LAST only reduces the redundancy by dropping off the longer overlapped seeds. However, the redundancy from the overlapped seeds that map to the reference sequence starting at different positions is not considered in this algorithm. Seeds redundancy could be eliminated by more sophisticated algorithms. However, eliminating seeds redundancy may be more time-consuming. LAST is a good tradeoff, removing part of the seed redundancy through a very simple approach.

AMAS [90] splits a read into several non-overlapping adaptive seeds. Specifically, the adaptive seeds are generated one by one through scanning the read base by base in the left-to-right direction. The first 10 bp in the read are used by default to initiate the scanning process. When the number of matches of a seed is less than a predefined frequency threshold, the seed is selected as an adaptive seed and a new seed scanning is initiated using the next 10 bp in the read. AMAS also makes an improvement on filtering the adaptive seeds, especially for the last seed in each read, which are usually shorter and map to much more locations than other seeds in the same read. In brief, AMAS only filters out last seeds of each read whose numbers of matches are higher than the predefined frequency cutoff and contribute to more than 95% of the candidate locations of their respective reads.

### 2.2. q-gram Filter

**Figure 3:**
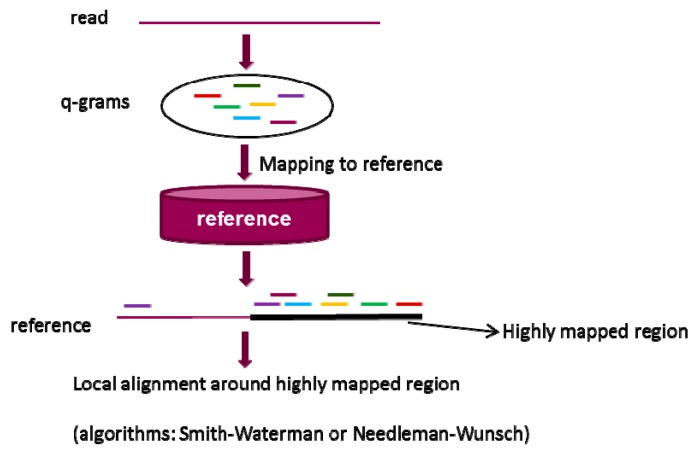
The overall workflow of q-gram filter in alignment. The strategy consists of three steps: (1) generation of q-grams from a read; (2) identification of highly mapped regions in a reference sequence through multiple q-grams mapping; and (3) local alignment of the read and highly mapped regions through standard dynamic programming algorithms based on Needleman–Wunsch (NW) [78] algorithm or Smith–Waterman (SW) algorithm [79].

Similar to seeds, q-grams are small fragments extracted from a read. q-gram filter alignment [91] is based on the hypothesis that two sequences should contain a certain number of q-gram if the edit distance between them is within a certain threshold. Herein, the edit distance [92] between two sequences is defined as the minimum number of editing operations (such as insertion, deletion, and substitution) that are needed to transform one sequence into the other. Figure 3 gives an overall flowchart of alignment based on q-gram filter strategy. In the first step, q-grams from a query read are generated and mapped to a reference sequence. This is a multiple-seeds mapping process. In the second step, the highly mapped regions are selected as candidates by an inverted index of grams, leading to a relatively larger memory usage. Lastly, the candidates are aligned to the read and the false positives are identified and discarded. The difference between q-gram filter and seed-and-extend is that q-gram filter based algorithms align a read to reference sequence by multiple seeds mapping without extension, while algorithms using seed-and-extend generate an alignment between a read and a reference sequence through mapping a single seed following by extension. Without extension process, q-grams filters based algorithms support more insertions and deletions in alignment than the algorithms using seed-and-extend strategy. 

SHRiMP (Short read mapping package) [61,62] was developed by University of Toronto and is a popular alignment software utilizing q-gram filter. It incorporates the concept of spaced seed in q-grams generation and mapping to improve alignment sensitivity. Many predetermined positions such as SNP (single nucleotide polymorphism) sites in a reference genome are not required to exactly match with reads during q-grams mapping. Candidate q-grams are selected using a predefined minimum of hits on a reference sequence. Number of hits of a q-gram on a reference sequence is used to estimate its mapping goodness. The candidate q-grams of each read are sorted according to their numbers of hits on the reference sequence. SHRiMP identifies candidates for each read by top ranking the q-grams using their numbers of hits on the reference sequence. The number of top hits used to identify candidate q-grams can be defined by users.

Hobbes [93] is an optimized q-gram filter based method for efficient read alignment. It improves both q-gram generation and candidate filtering. A basic q-gram method usually generates numerous overlapped small q-grams for a read and thus needs intensive CPU calculation and large memory usage. Hobbes uses non-overlapping q-grams to optimize the number of q-grams based on a paradigm similar to the pigeonhole principle used in k-mer inexact match seed. The rationale behind this algorithm is that a read within an edit distance *d* to a reference sequence must contain at least *n* q-grams from *d* + *n* non-overlapping q-grams of the reference sequence. In candidate selection, Hobbes uses two approaches to rigorously filter the highly mapped regions of non-overlapping q-grams in the reference sequence for the read. At first, it filters the candidate q-grams based on the edit distance between corresponding reference sequence and the neighboring sequence of the mapped q-gram. The hypothesis is that if the neighbor of a matched q-gram has a large edit distance to the corresponding sequence in the read, the candidate q-gram is probably not a true match. The difference in frequency between the highly matched regions by overlapping q-grams and the read for the four bases (A, T, G, and C) is another filter to remove invalid candidate q-grams for further alignment evaluation.

## 3. Conclusions

The rapid development of next-generation sequencing technologies provides a promising opportunity to extend the capability of biomarker discovery in precision medicine. How to efficiently and correctly map millions of short reads to a reference genome is one of the major challenges in NGS data analysis. More specifically, speed and sensitivity are the two major concerns in current alignment algorithms, no matter which seed-and-extend or q-gram filter strategy is utilized. In this review, we have summarized the often-used alignment algorithms and discussed the approaches to achieve an optimized tradeoff between speed and sensitivity. Generally, higher sensitivity would be achieved by using shorter seeds or grams with more mismatches allowed, while alignment speed can be increased by optimizing seed generation or filtering seeds that most likely fail to extend. This review is expected to facilitate understanding of the alignment algorithms and their algorithmic parameters.

## 4. Further Perspectives

Many state-of-the-art software packages have already made great progresses in achieving an optimized tradeoff between speed and sensitivity. However, there are still some rooms for improvement.

One of the remaining challenges in reads alignment is how to align the reads that can be mapped to multiple repeated regions in a reference genome. According to the statistics on human reference genome version hg19, approximately 50% of the human genome has repeats [94]. Especially, some copies of the repeats are not the same but slight variants. This inevitably causes ambiguities in reads mapping. Usually, seeds or q-grams of reads are not specific and mapping may be very slow in the repeated regions. Currently, three simple methods have been used to improve alignment speed in repeated regions. The first method is to discard all seeds or q-grams that map to repeated regions. The second one is to randomly select one of the best alignments or report all of them. The last one is to select a number of top alignments. Obviously, ignoring all of the reads or part of reads mapped in the repeats may miss some important variants. In addition, the “best alignment” identified by an alignment software package in such regions may not always be correct, especially when a SNV or a small indel truly occurs in the repeat region. Nathan [45] first proposed a probabilistic model base on quality scores to align reads in repeated regions. Further efforts are still needed to improve alignment of reads in repeated regions of a reference genome.

Another challenge is to develop alignment algorithms for extremely long reads. Although most current NGS platforms produce short reads with length around 2 × 150 bp, there is no doubt that extremely long reads generated by so-called “third generation sequencing” platforms would be more and more promising and provide fundamentally more information than short reads. A finite coverage with short reads is not enough for deciphering a complex genome, especially for the regions where no or very few reads are mapped. A few long reads in the right spot may be able to identify the genetic variants. However, the error rate in long read platforms is a major concern in application of long reads. Most current algorithms are designed for alignment of short reads. Many parameters in the algorithms for alignment of short reads would not be appropriate for alignment of long reads. For example, only the first 28 bp in each read was supported for at most three mismatches by default in Bowtie. Therefore, the extension process would be extremely long if a small k-mer seed was used to initiate the alignment of a long read. It is expected that new alignment algorithms will be exclusively designed for long reads, with consideration of their specific properties.

Integration of additional genetic information into sequence alignment will be a focus in the future. Currently, the majority of the alignment algorithms were designed to align reads to a single reference genome without consideration of the genetic variations in the reference genome. The assumption behind current algorithms is that the reference genome is highly similar to the genome sequenced and provides comprehensive enough genetic background. However, precision medicine needs genetic difference among individuals, thus such hypothesis is challenged. Human reference genome may not provide the most comprehensive genetic variations. A study on the novel assembly of an Asian and an African genome revealed ~19–40 Mb of novel sequences that were missed in the human reference genome [95]. In order to achieve more sensitivity and accuracy in alignment, genetic variation information, such as the 1000 genomes project [25] and the structural variation information in cancer from TCGA [96], should be integrated into alignment algorithm development in the future.
